# Plant-Growth Promotion and Biocontrol Properties of Three *Streptomyces* spp. Isolates to Control Bacterial Rice Pathogens

**DOI:** 10.3389/fmicb.2019.00290

**Published:** 2019-02-25

**Authors:** Zulma Rocío Suárez-Moreno, Diana Marcela Vinchira-Villarraga, Diana Isabel Vergara-Morales, Leonardo Castellanos, Freddy A. Ramos, Corrado Guarnaccia, Giuliano Degrassi, Vittorio Venturi, Nubia Moreno-Sarmiento

**Affiliations:** ^1^Investigación y el Desarrollo, Biocultivos S.A., Bogotá, Colombia; ^2^Instituto de Biotecnología, Universidad Nacional de Colombia, Bogotá, Colombia; ^3^Departamento de Química. Universidad Nacional de Colombia, Bogotá, Colombia; ^4^Biotechnology Development Unit, International Centre for Genetic Engineering and Biotechnology, Trieste, Italy; ^5^Bacteriology and Plant Bacteriology Group, International Centre for Genetic Engineering and Biotechnology, Trieste, Italy

**Keywords:** Streptomyces, biological control, plant growth promotion, Burkholderia glumae, streptothricins

## Abstract

Bacterial Panicle Blight caused by *Burkholderia glumae* is a major disease of rice, which has dramatically affected rice production around the world in the last years. In this study we describe the assessment of three *Streptomyces* isolates as biocontrol agents for *B. glumae*. Additionally, the presence of other plant-growth promoting abilities and their possible beneficial effects upon their inoculation on rice plants was evaluated as an ecological analysis for their future inoculation in rice crops. Two isolates (A20 and 5.1) inhibited growth of virulent *B. glumae* strains, as well as a wide range of bacterial and fungal species, while a third strain (7.1) showed only antifungal activity. *In vitro* tests demonstrated the ability of these strains to produce siderophores, Indoleacetic acid (IAA), extracellular enzymes and solubilizing phosphate. Greenhouse experiments with two rice cultivars indicated that *Streptomyces* A20 is able to colonize rice plants and promote plant growth in both cultivars. Furthermore, an *egfp* tagged mutant was generated and colonization experiments were performed, indicating that *Streptomyces* A20 –GFP was strongly associated with root hairs, which may be related to the plant growth promotion observed in the gnotobiotic experiments. In order to characterize the antimicrobial compounds produced by strain A20 bacteria, mass spectrometry analyses were performed. This technique indicated that A20 produced several antimicrobial compounds with sizes below 3 kDa and three of these molecules were identified as Streptotricins D, E and F. These findings indicate the potential of *Streptomyces* A20 as a biocontrol inoculant to protect rice plants against bacterial diseases.

## Introduction

Bacterial Panicle Blight (BPB) caused by *Burkholderia glumae* is a bacterial disease of rice with an increasing occurrence in South America since its first detection in 2007 (Correa et al., 2007; Castillo et al., 2011; Ham et al., 2011). This bacterial pathogen was first detected in Japan (Goto and Ohata, 1956; Tanii et al., 1976) and is currently widespread around the world. In the last 20 years, *B. glumae* has shown a significant occurrence in rice-growing countries in Latin America and United States causing grain rot and seedling rot of rice (Zeigler and Alvarez, 1987; Correa et al., 2007; Nandakumar et al., 2007; Diago et al., 2009; Nandakumar et al., 2009). *B. glumae* causes bacterial wilt in a wide variety of plant hosts, but disease in rice is the most studied due to the dramatic economic effects of BPB in rice yields (Jeong et al., 2003). Major symptoms of this BPB include panicle blight, seedling blight, and sheath rot, with a linear lesion extending downward from the leaf blade collar forms on the flag leaf. Affected panicles may have one or all of their florets blighted with grains not filling or aborting, which causes typical upright brown panicles due to the failure of grain filling (Goto and Ohata, 1956; Kurita and Tabei, 1967; Goto et al., 1987; Zeigler and Alvarez, 1987).

In Latin America and the United States, rice yield reductions due to BPB have reached 75% in severely infested fields as a result of a reduction in grain weight, sterility of florets and inhibition of seed germination (Correa et al., 2007; Nandakumar et al., 2007, 2009; Diago et al., 2009). In addition to the problems due to BPB, losses due to SBR caused by the phytopathogen *Pseudomonas fuscovaginae* have increased dramatically in rice-producing countries like Indonesia, Malaysia, and Colombia, with losses up to 76% (Razak et al., 2009). The occurrence of both pathogens has been favored by changes in climatic conditions and it is believed that these diseases may occur more frequently in tropical and semi-tropical countries.

The main current approaches to control *B. glumae* include rice breeding to obtain tolerant rice varieties (Mizobuchi et al., 2013) and isolation and identification of virulent strains, which have been analyzed at genomic level to identify putative virulence genes differing among them (Francis et al., 2013; Fory et al., 2014). Also, improvement of diagnostic tools for achieving detection at early stages of rice development, and intense diffusion and training of farmers in symptoms-detection in the fields have been used as an approach to diminish the incidence of *B. glumae* (Sayler et al., 2006; Kawanishi et al., 2011). Furthermore, recently transcriptome studies for the pathosystem rice-*B. glumae* have contributed to an understanding of the gene expression of this pathogen along rice development (Kim et al., 2014; Magbanua et al., 2014). Nonetheless, these approaches are not always efficient, considering that virulent strains are polymorphic and have developed resistance to chemical treatments (Hikichi et al., 2001; Karki et al., 2012).

In spite of the dramatic losses due to *P. fuscovaginae* and *B. glumae*, no biological or chemical approach has proven to be successful for controlling them in Latin America, and consequently, sustainable alternatives for reducing these two bacterial diseases of rice in the region are urgently needed. A possible alternative is a biocontrol approach, which involves the use of disease-suppressive microorganisms to control pathogens and improve plant health (Handelsman and Stabb, 1996). Major aspects of biocontrol are bioprospecting for new active isolates as well as understanding the mechanisms of pathogen antagonism for their future improvement and broader use.

Many *Streptomyces* strains are considered biocontrol agents, since they produce a wide range of antimicrobials, can persist in harsh environments, and efficiently colonize the rhizosphere of different plant species including rice (Qin et al., 2011; Kinkel et al., 2012). Furthermore, *Streptomyces* are able to elicit induced resistance, as it has been described before (Conn et al., 2008; Kurth et al., 2014). Because of these features, it is not surprising that diverse *Streptomyces* strains had been studied to control fungal and bacterial diseases of rice like Bacterial Leaf Blight caused by *Xanthomonas oryzae*, however very few *Streptomyces* are currently being developed as biocontrol products.

Research and development of biocontrol products commonly focuses on four major points: (1) studying the biocontrol traits for microbial agents, (2) assessment of the plant–microorganism interaction, (3) monitoring the beneficial and ecological effects of inoculation of each agent in the rhizosphere, and finally (4) verifying proper release of the microbial agents upon inoculation of crops with formulated products (Herrmann and Lesueur, 2013; Miransari, 2013). In this study, we describe the isolation and characterization of three *Streptomyces* isolates and assess their potential as biocontrol agents in order to explore their interaction and possible beneficial effects upon inoculation of rice plants. Colonization and plant growth promotion experiments were performed in sterile and non-sterile conditions as an approach to evaluate the ecological effects of their use in rice fields, and the colonization patterns were visualized microscopically for one isolate.

## Materials and Methods

### Isolation and Identification of *Streptomyces*-Like Isolates

Forty-five samples of rhizospheric soils were collected from rice-cultivated fields in Tolima (Colombia). In order to conduct this research, the ANLA (Autoridad Nacional de Licencias Ambientales) and the Ministry of Environment (Ministerio de Ambiente y Desarrollo Sostenible) were requested for permission to collect samples and study the recovered bacteria.

Briefly, plants roots were removed from the soil and subsequently handshaked for 10 min to remove bulk soil. The remaining adhering soil was considered as rhizospheric soil, and was collected by handshaking roots for 10 min in a sterile plastic bag. Rhizospheric soil samples (4–5 g for each) were then mixed with 1 g of CaCO_3_. Samples were further dried at 45°C for 1 h, as described previously (Gurung et al., 2009). Actinobacteria were subsequently isolated by spread plate technique following the serial dilution of soil samples on starch casein agar (Starch 10 g/L, Casein 1 g/L, K_2_HPO_4_ 0.5 g/L, Agar 13 g/L) and incubated at 30°C for a week. In order to select *Streptomyces-*like bacteria, microscopic and macroscopic characteristics of the obtained colonies were assessed resulting in a total of 60 isolates, which were further purified and sub-cultured. Single colonies were characterized by their colony morphology in International Streptomyces Project media (ISP2, ISP3, ISP4), Nutrient agar and Mueller Hinton Agar (Supplementary Figure S1). Catalase, oxidase and their carbon source utilization were tested, as suggested for *Streptomyces*-like bacteria (Shirling and Gottlieb, 1968; Goodfellow, 2012).

### Antimicrobial Activity Assays

Actinobacteria strains were grown for 5 days in M3.7 liquid media (glucose 5 g/L, Yeast Extract 5 g/L, (NH_4_)_2_SO_4_ 5 g/L, Corn gluten 5 g/L, CaCO_3_ 2 g/L, NaCl 2 g/L, FeSO_4_ 1 mg/L, starch 10 g/L pH 7.2). Five-day cultures were used for antimicrobial tests. Antibacterial activities against 21 strains from 15 species (Supplementary Table S1) were determined by using the Kirby-Bauer agar well-diffusion method, modified from the CLSI 2011 guidelines (Clinical Laboratory Standards Institute, 2011). Briefly, each strain to be tested was grown overnight in LB media, and its OD_600_ was determined. Bacterial absorbance was then adjusted to 0.25, and a cotton swab was fully immersed in each bacterial dilution. The inoculated swab was used to spread the entire surface of a Mueller Hinton Agar plate, containing 25 mL of agar five times. Seven mm (diameter) wells were perforated in the agar, and 50 μL of each actinobacterial culture were poured into the well. Plates were subsequently incubated at 30 or 37°C (according to the best temperature for each bacteria to be tested). Inhibition zones were measured after 24 h of incubation. Antibacterial assays were also performed against a collection of 48 *B. glumae* isolates recovered from rice plants exhibiting symptoms of BPB (kindly provided by FEDEARROZ-Colombia).

Antifungal activities against 9 fungal plant pathogens (Supplementary Table S1) were measured as described previously (Kanini et al., 2013). For this assay, fungal strains were grown in Potato Dextrose Agar (PDA) plates for 3 days at 25°C, and two 6 mm disks of mycelium from each phytopathogenic fungi were then placed in opposites edges of a new PDA plate. Following fungal inoculation, two 6 mm wells were open in opposite sides of the PDA plate, containing 50 μL of a 5-day culture for each Actinobacteria strain tested. The plates were incubated at 28°C for 5 days and antagonistic activity was estimated by measuring the growth inhibition zone.

### Taxonomical Identification and Multilocus Sequence Analysis (MLST)

Three strains were selected based on their wide range of antimicrobial activity. Genomic DNA was isolated by using the Salting-out procedure described previously (Pospiech and Neumann, 1995), with few modifications. Briefly, single colonies of each isolate were grown in 30 mL Tryptic Soy Broth, with shaking at 30°C for 48 h. Cells were harvested by centrifugation and washed with 10% sucrose, and suspended in 5 ml of STE Buffer (75 mM NaCl, 25 mM EDTA pH 8.0, 20 mM Tris-HCl pH 7.5), containing heat-treated pancreatic RNase A at 10 mg/mL. Hundred microliters of lysozyme (50 mg/mL) were added to the mixture and incubated for 1 h at 37°C, with gentle tapping at intervals. This was followed by the addition of 140 μL of proteinase K (20 mg/ml) and 600 μL of 10% SDS, with further incubation at 55°C for 2 h. Two mL of 5M NaCl were added and mixed by inversion until the temperature of the suspension reached the 37°C. The samples were cooled to room temperature and extracted twice with 5 ml of chloroform. Aqueous fractions were separated by centrifuging at 10000 *g* for 10 min at 4°C, and genomic DNA was precipitated by adding one volume of isopropanol, mixed by inversion and centrifuged 15 min at 15000 rpm. The obtained DNA pellet was rinsed with 70% ethanol, air-dried and suspended in 100 μL of DNAse-free sterile water.

Taxonomical identification was first approached by PCR amplification, sequencing, and analysis of the entire 16S rRNA locus by using primers 16S_A and 16S_B described by Cui et al. (2001), and the cleaned PCR products were directly sequenced using universal primers 27F, 500F, 818R and 1492R (Supplementary Table S1). Closely related sequences were obtained from RDP (Ribosomal Database Project) (Cole et al., 2014) and EZTaxon (Kim et al., 2012). Obtained sequences were imported into MEGA6 software and aligned with ClustalW for phylogenetic analysis (Thompson et al., 1994).

Multilocus Sequence Typing was used to elucidate a further taxonomical affiliation (Guo et al., 2008; Rong et al., 2009). Briefly, five loci (*gyrB, atpD, recA trpB*, and *rpoB)* were PCR amplified and purified by using the EuroGOLD Gel extraction Kit (EuroCLONE, Italy). Each fragment was then sequenced by using primers described previously (Guo et al., 2008), and listed in Supplementary Table S1. The sequences for all loci for each strain were concatenated head to tail in frame and exported in FASTA format. Sequences were aligned using CLCbio Mainworkbench 6.9.1 (CLC-Bio Qiagen) and MEGA 6.0 (Tamura et al., 2011). A phylogenetic tree was constructed from the concatenated sequences of all six loci, and representative *Streptomyces* strains available in the *Streptomyces* MLST database (Jolley and Maiden, 2010)^1^ Maximum likelihood algorithm trees were generated with MEGA based on the Tamura-Nei model (Tamura and Nei, 1993), and a tree with the highest log likelihood was generated calculating the percentage of trees in which the associated taxa clustered together. Initial trees for the heuristic search were obtained automatically by applying Neighbor-Join and BioNJ algorithms (Gascuel, 1997) to a matrix of pairwise distances estimated using the Maximum Composite Likelihood (MCL) approach, and then selecting the topology with superior log-likelihood value.

### *In vitro* Assessment of Plant Growth Promotion (PGP) Traits

Biological nitrogen fixation, phosphate solubilization, indoleacetic acid production (IAA), ACC deaminase and extracellular enzyme production were evaluated. For this purpose, each isolate was grown in 100 mL M3.7 medium for 5 days, and each assay was performed with three independent cultures for each strain.

#### Biological Nitrogen Fixation

To test biological nitrogen fixation, Acetylene Reduction Assay (ARA) was performed as described elsewhere (Hardy et al., 1968). To this purpose, 100 μL of washed cells were inoculated into 17 mL of semisolid NFb medium in 25 mL serum bottles with a cotton cap, and cultures were maintained at 30°C, without agitation (Dobereiner, 1995). After 5 days, 2 mL of acetylene were injected into the flask and a rubber stopper was used to replace the cotton cap. The acetylene and ethylene concentrations of each sample were determined by gas chromatography, using a chromatograph Varian 3400-G-crom, equipped with a flame ionization detector and a capillary column (Hayesep Porapak N 80/100 column; 6′ X 1/8″). Ethylene and acetylene estimation was done by integrating the area under the curve for each compound, at retention times of 1.417 and 2.40 min, respectively.

#### Phosphate Solubilization

Phosphate solubilizing abilities were qualitatively determined by inoculating single colonies of each strain in National Botanical Research Institute’s Phosphate growth medium (NBRIP) (Nautiyal, 1999) and SRS media (Sundara-Rao and Sinha, 1963). Plates were incubated at 30°C for 5 days, and colonies with a clear orange halo in NBRIP media and/or a purple color in SRS media were marked positive for phosphate solubilization. A quantitative determination was performed by growing each strain in 100 ml of NBRIP liquid media supplemented with 5 g/L CaCO_3,_ for 5 days. Subsequently, the solubilized phosphate present in the supernatant was measured with the Spectroquant Phosphor Kit (Merck).

#### Siderophore Production

Siderophore production was determined in Chrome Azurol Blue agar (Schwyn and Neilands, 1987), modified to use KOH to adjust the pH, as suggested previously (Mahmoud and Abdallah, 2001).

#### ACC Deaminase Production

1-aminocyclopropane-1-carboxylate (ACC) deaminase production was determined by checking the ability of each strain to use ACC as sole nitrogen source. For this purpose, single colonies were inoculated in Dorwin-Foster media (DF) supplemented with 3 mM ACC as sole nitrogen source, or with 2 g/L (NH_4_)_2_SO_4_ as growth control (Belimov et al., 2001; El-tarabily, 2008). In each case, 2 μL of a 10^6^ CFU/mL culture, were spotted on the surface of a DF plate with or without N source, and growth was evaluated after 5 days at 30°C, and *Burkholderia unamae* MT1 641T was included as a positive control. An absence of phenotypic changes in growth or sporulation of the isolates in the medium supplemented with ACC in comparison with an (NH_4_)_2_SO_4_ supplemented medium were considered as indicators of the capacity of the microorganisms to use ACC as a source of nitrogen mediated by the production of ACC-deaminase.

#### Indoleacetic Acid

For the quantitative determination of Indoleacetic acid (IAA) and IAA-like molecules, the colorimetric Salkowski’s assay was performed (Tang and Bonner, 1948). For this purpose, each *Streptomyces* strain was grown in 20 mL Tryptic Soy Broth (TBS) supplemented with 60 mM tryptophan. After 4 days, 1 ml of Salkowski reagent (Glickmann and Dessaux, 1995) was pipetted into test tubes containing 1 ml of culture spent supernatant. The tubes containing the mixture were gently vortexed and left for 30 min for development of color at room temperature (26 ± 1°C). The intensity of the color was spectrophotometrically determined at 535 nm.

#### Extracellular Enzyme Production

Extracellular enzyme production was evaluated *in vitro*. Cellulolytic activity was evaluated by spotting 2 μL of 5-day old cultures on the surface of CMC-media plates, in which CMC was the sole carbon source. Spots were incubated at 30°C for 4 days, and then strains were assayed for their ability to degrade CMC by flooding each plate with a 0.2% Congo red solution for 15 min, followed by washing with 1 M NaCl. Activity halos dimensions were then measured from the border of the colony to the outer edge of the halo. Similarly, ligninolytic activities were determined in guaiacol containing media and positive activity was scored when a red halo was formed around colonies, due to the oxidation of the substrate to tetraguaiacol, as described previously (Nagadesi and Arya, 2012).

### Plant-Growth Promotion Experiments

#### Gnotobiotic Experiments

Gnotobiotic inoculation experiments were performed in rice cultivars FEDEARROZ 733 (F733) and FEDEARROZ 60 (F60). For this purpose, rice seeds were surface sterilized and pre-germinated in 1% agar, for 5 days in complete darkness (Mattos et al., 2008). Each seed was then transferred into glass flasks (15 cm × 4 cm) with 40 mL of semisolid Hoagland media previously inoculated with 10^3^ CFU/mL of each strain. To this end, each strain was grown in M3.7 media, and cells were suspended in sterile saline solution and added to the semi-solid medium to reach a final concentration of 10^3^CFU/mL, before solidification of the medium.

Plantlets were then transferred to a greenhouse (Relative humidity 43% ± 8.6, Average Temperature 24°C ± 5.2, natural light). *Azotobacter chroococcum* (APFN010) and *Azospirillum brasilense* (AZPP010) strains (10^5^ CFU/mL) were used as positive controls, and sterile water was used as negative control. Each treatment included two biological replicates (two independent colonies for each bacterial strain), and 10 plants were inoculated with each inoculum. The entire experiment was performed twice, and biometric properties (root and shoot fresh weight, plant dry weight, and length) were measured 15 days post-inoculation.

#### Soil Inoculation Experiments

Inoculation experiments were also performed in sterile and non-sterile soil using cultivars F733 and F60 with pregerminated seeds, as described above. For these assays, 10 mL from a 10^8^ CFU/mL inoculum were mixed with 250 *g* of sterile soil, to reach a concentration of 10^5^ CFU/g. Subsequently, pre-germinated seeds were planted inside the pot and plants were kept in the greenhouse at 25°C for 31 days post-inoculation. *A. chroococcum* (APFN010) and *A. brasilense* (AZPP010) strains were used as positive controls, and sterile water was used as negative control. Each treatment included two biological replicates (two independent colonies for each bacterial strain), and 10 plants were inoculated with each inoculum. Biometric properties (root and shoot fresh weight, plant dry weight and length) were measured 31 days post-inoculation. Dry weight for each plant was determined after drying the plant material at 50°C, until obtaining a constant weight.

#### Plant Colonization Experiments

To assess the ability of the three *Streptomyces* for rice plant colonization, two independent assays were performed under gnotobiotic and soil conditions as described before, using in each case three biological replicates for each strain, with five plants per replicate. Bacterial strains were re-isolated from the rhizoplane by cutting and washing 2 cm segments of each plant root and placing the excised root segments on the surface of ISP3 plates. Roots of the soil experiment were placed on ISP3 medium supplemented with nalidixic acid (20 μg/ml) and nitrofurantoin (10 μg/ml) to inhibit the growth of other soil microorganisms. The identity of colonies recovered from the roots was verified from their phenotype and their antimicrobial profile.

To verify endophytic colonization, inoculated plants were surface sterilized with 1% sodium hypochlorite and 70% ethanol for 4 min each, followed by several washes with sterile water. Each plant was then excised in the root, plant shoot and leaves, and each part was macerated with a sterile pestle. Each suspension was then serially diluted and plated in ISP3 plates. Surface sterilization was regarded as optimum when no growth was observed: (1) after placing the processed plants on ISP3 plates, and (2) after plating 100 μL from the last wash in ISP3 (see more details from the experimental set-up in Supplementary Figure S2).

Finally, to test the persistence and viability of isolates in the soil, rhizospheric soil portions were sampled from each treatment. 1 g of each sample was diluted in Tween 80 (0.1% v/v) and plated in ISP3 medium supplemented with nalidixic acid and nitrofurantoin. Results were evaluated after 4 days incubation at 30°C and reported as CFU/g soil rhizosphere.

### Fluorescence Microscopy

An *egfp* tagged mutant from *Streptomyces* sp. A20 was generated by intergeneric conjugation with *Escherichia coli* S17-1 λ pir harboring pIJ8641 plasmid, (kindly provided by M. Bibb from the John Innes Centre-Norwich, United Kingdom) (Sun et al., 1999; Kieser et al., 2000; Willey et al., 2007), and transconjugants were selected into Apramycin ISP3 plates (100 μg/ml). Fluorescent transconjugants were further selected for gnotobiotic inoculation experiments as described before, using either semisolid Hoagland or sterile vermiculite supplemented with Apramycin (100 μg/ml). For these experiments, 15 plants were inoculated with A20pIJ8641, non-tagged *Streptomyces* A20, and a non-inoculated set of plants was included as a control. To assess the colonization process three roots were evaluated for each treatment at days 1, 2, 3, 10, and 15 days post inoculation. In each evaluation day, the roots were excised from the matrix, washed three times with sterile water and cut in 3 mm × 3 mm fragments. Observations were performed in an Olympus BX41 Microscope with a CCD Infinity 3 Camera (dichroic mirror DM500; excitation filter BP460-490 and barrier filter BA520IF).

### Purification and Characterization of Antimicrobial Compounds

*Streptomyces* A20 was grown in 80 L M3.7 media in a 100 L stirrer tank bioreactor with constant shaking (40 rpm) at 25°C. After 4 days, 1 L of culture was centrifuged and spent supernatants were filter-sterilized using a 0.22 μm membrane. Bioassay-guided fractionation of A20 fermentation sterile supernatants was then performed by a sequential partition with ethyl acetate (2 × 500 mL), followed by butanol (4 × 500 mL) extraction (Supplementary Figure S3), to yield organic (EtOAc), butanolic and residual aqueous fraction (ABP fractions). Each fraction was evaporated to dryness and suspended into sterile water to be subjected to antibacterial assays. Active fractions were first purified by ultra-filtration by using 1, 3, and 10 kDa Amicon filters (Millipore).

Several chromatographic techniques were used to purify antimicrobial compounds produced by strain *Streptomyces* A20. Briefly, residual aqueous fractions from butanol extraction were dried and suspended into buffer A (sodium acetate 0.05 M pH 5.5) to be loaded into a strong anion exchanger column, Q Sepharose Fast Flow, previously equilibrated and washed with buffer A, using a BioRad Biologic Duoflow chromatography system. Compounds bound to the column were eluted with a linear gradient of increasing NaCl concentration (0–1M). Antimicrobial compounds present in the flowthrough, were purified by loading it into an S Sepharose^TM^ Fast Flow column (cation exchange chromatography), and antimicrobial compounds were eluted again with a gradient of increasing NaCl concentration. Antimicrobial fractions were then suspended into 20 mM ammonium acetate, containing 0.1% heptafluorobutyric acid (HFBA), and injected onto a semipreparative C18 reversed phase column (1x 25 cm). Three fractions were recovered due to their antibacterial activity and were further analyzed by LC-MS.

Samples for LC-MS were dissolved in 90% methanol and 10 μl injected onto a Gemini C18 column (2 × 150 mm, 5 μm; Phenomenex, Torrance, CA, United States) and separated using a gradient from water to 90% acetronitrile mobile phases containing 0.1% formic acid at 0.2 ml min^-1^. The column effluent was delivered to an ESI ion trap mass spectrometer (Amazon SL, Bruker Daltonics, Bremen, Germany) and mass spectra collected in positive ion mode in the range 200–2000 m/z.

### Statistical Analysis

Most experiments were performed at least three times and means are given. Statistical analyses included unpaired *t*-tests and ANOVA with Dunnett’s post-test and were performed with PRISM 4.0 software (GraphPad Software, San Diego CA, United States). A *P*-value of < 0.05 was considered significant.

### DNA Sequencing and Nucleotide Sequence Accession Numbers

16S rRNA nucleotide sequences for strains *Streptomyces* A20, 5.1, 7.1 and *Streptomyces racemochromogenes DSM* 40194 were deposited in GenBank/EMBL/DDBJ, under the accession numbers KP082880, KP082881, KP082882, and KP082883.

## Results

### Isolation and Identification of *Streptomyces* Strains With Anti-bacterial Activity From Rice Cultivated Soils

Sixty actinobacterial-like isolates were obtained from Colombian soils upon enrichment with CaCO_3_ however, only three isolates denoted as A20, 5.1 and 7.1 showed antimicrobial activity against either two plant pathogenic bacterial strains of *B. glumae* and *P. fuscovaginae*, or fungal phytopathogens. Strains A.20 and 5.1 showed both antibacterial and antifungal activity, whereas strain 7.1 showed only antifungal activity (Table 1).

**Table 1:** Antibacterial and antifungal activity of *Streptomyces* A20, 5.1, and 7.1.

Strain	A20	5.1	7.1
**Antibacterial activity**			
*Acidovorax avenae* CIAT 4008-2	++	++	–
*Acinetobacter baumanii* ATCC 19606	+	–	–
*Bacillus* sp. *C636*	+	+	–
*Bacillus subtilis* ATCC 21556	+	+++	–
*Burkholderia glumae* 320012	+++	++++	–
*Burkholderia glumae* AU6208	+++	+++	–
*Burkholderia glumae* CIAT 4026	++	+++	–
*Burkholderia gladioli* CIAT 3704-1	+	+++	–
*Burkholderia gladioli* CIAT 3962	+	++	–
*Chromobacterium violaceum* ATCC 31532	++	++++	–
*Enterobacter aerogenes* 7ARG	+	–	–
*Escherichia coli DH5*α	+++	++	–
*Escherichia. coli* ATCC 23724	+++	–	–
*Escherichia coli* ATCC 25922	++	+	–
*Klebsiella pneumonie* ATCC 700603	+	++	–
*Pseudomonas putida* F117 pKRC12	+++	–	–
*Pseudomonas aeruginosa* ATCC 27853	–	++	–
*Pseudomonas fuscovaginae* CIAT 3638-19	+	++++	–
*Pseudomonas fuscovaginae* CIAT 3668π3	+	++	–
*Staphylococcus aureus* ATCC 25923	+	+++	–
**Antifungal activity**			
*Fusarium* sp. DC9	+	+	–
*Fusarium* sp. *DC 13B*	+	+	–
*Fusarium oxysporum*	+	+	+
*Gaeumannomyces* sp.	+	+	+
*Phomopsis* sp.	+	+	–
*Ulocladium* sp.	+	+	+
*Rhizoctonia solani*	+	+	–
*Colletotrichum* sp. 24C	+	+	+
*Colletotrichum* sp. *26B*	+	+	+


*Results are means of three inhibition halo-diameter (mm) measurements in three independent replicates for each strain tested. Antibacterial activity: (-) No inhibition detected, (+) 5–10 mm, (++) 11–15 mm, (+++) 16–20 mm, (++++) > 20 mm. Antifungal activity: (-) No inhibition detected, (+) Inhibition zone detectable.*

**FIGURE 1:**
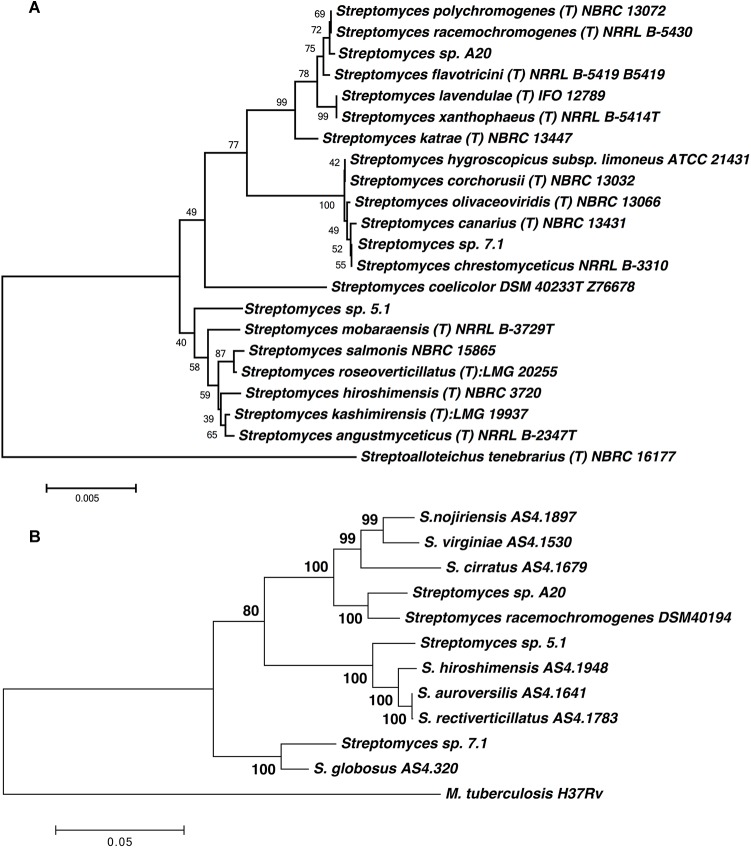
**(A)** Phylogenetic tree based on 16S rRNA sequences of the *Streptomyces* strains. The evolutionary history was inferred using the Neighbor-Joining method (Saitou and Nei, 1987). The tree is drawn to scale, with branch lengths in the same units as those of the evolutionary distances used to infer the phylogenetic tree. Numbers at branch nodes are bootstrap values. Sequence from *Streptoalloteichus tenebrarius* NBRC 16177 was included as outgroup. **(B)** Phylogenetic tree based on MLST analyses from five loci (*gyrB, atpD, recA trpB*, and *rpoB*). The tree was drawn to scale using Maximum Likelihood method and Tamura-Nei model, with branch lengths measured in the number of substitutions per site. The analysis involved 12 nucleotide sequences. All positions containing gaps and missing data were eliminated. There were a total of 2896 positions in the final dataset.

Biochemical tests indicated that these three strains were catalase positive; oxidase negative, and their carbon source analysis indicated that all strains were able to metabolize glucose, but were unable to use rhamnose, arabinose, xylose or mannitol as carbon source (Table 2). Taxonomical analyses derived from 16S rRNA sequencing confirmed that all three strains belong to the genus *Streptomyces* and similarity calculations of the 16S rRNA locus, based on Maximum Likelihood and Neighbor Joining analyses indicated that the closest relatives for strain A20 were *S. racemochromogenes/S. polychromogenes* and *S. flavotricini* with similarity values of 99.93 and 99.8 % respectively, placing this strain within the previously described clade 38 (Labeda et al., 2012). A similar analysis located strain 7.1 within clades 2 and 71 with similarity values of 99.89 and 99.79% to *S. chrestomyceticus*, *S. corchorusii/S. canarius.* Strain 5.1 was related to the S. *roseoverticillatus* clade 65, having 98.9% similarity to *Streptomyces kashimirensis* and *S. salmonis* (Figure 1A).

**Table 2:** Biochemical characterization of Streptomyces A20, 5.1 and 7.1.

Strain	Cat	Oxi	Glu	Suc	Fru	Rha	Ara	Ino	Xyl	Man
A20	+	–	+	–	–	–	–	–	–	–
7.1	+	–	+	+	+	–	–	–	–	–
5.1	+	–	+	–	–	–	–	+	–	–


*(-) No sugar fermentation detected or negative reaction. (+) sugar fermentation detected or positive reaction. Cat, Catalase. Oxi, Oxidase. Glu, Glucose. Suc, Sucrose. Fru, Fructose. Rha, Rhamnose. Ara, Arabinose. Ino, Inositol. Xyl, Xylose. Man, Mannitol.*

In order to improve the taxonomical identification, MLST was performed for all three strains, and type strain *S. racemochromogenes* DSM 40194. MLST analyses for 12 strains, including the closest neighbors for each isolate revealed that all six loci have unique sequences compared to all available allele types in the MLST *Streptomyces* database. MLST profiles for strains 5.1 and 7.1 defined these two isolates as independent entities, and MLST profiles for close neighbors these two should be obtained to improve their taxonomical identification.

All loci obtained for strain A20 were highly similar to those obtained for strain *S. racemochromogenes* DSM 40194, and concatenated loci for both strains shared 97.2% identity at sequence level, however evolutionary distance calculated with Kimura 2 parameters, was above the species-definitive MLSA distance of 0.007 (0.028) (Figure 1B) (Rong and Huang, 2012; Labeda et al., 2014). Further studies may be required to generate MLST profiles for other type strains from this cluster in order to verify these results and define if strain A20 corresponds to a known species or may be described as a new species.

### *Streptomyces* A20, 5.1, and 7.1 Exhibit a Wide Range of Antimicrobial Activity

**FIGURE 2:**
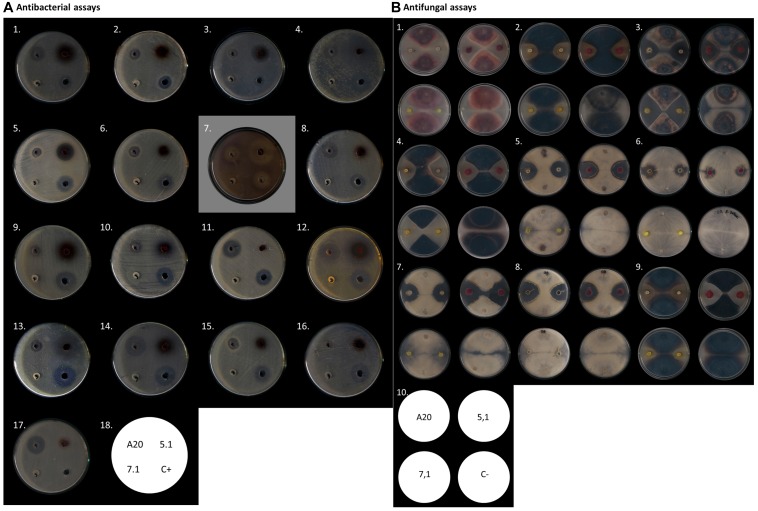
Antimicrobial activity of *Streptomyces* A20, 5.1, and 7.1. **(A)** Antibacterial assays by Kirby- Bauer well diffusion test. In the figure from left to right: (1) *A. avenae* CIAT 4008-2, (2) *A. baumannii* ATCC19606, (3) *S. aureus* ATCC 25923, (4) *B. glumae* CIAT 4026, (5) *B. subtillis* ATCC 21556, (6) *Bacillus* C636, (7) *C. violaceum* ATCC 31532, (8) *E. coli* ATCC12167, (9) *B. gladioli* CIAT 3962, (10) *B. gladioli* CIAT 3704-1, (11) *E. coli* ATCC23724, (12) *B. glumae* 320012, (13) *P. fuscovaginae* CIAT 3638-19, (14) *E. coli* DH5α, (15) *K. pneumoniae* ATCC 700603, (16) *P. aeruginosa* ATCC 27853, (17) *P. putida* F117. (18) Assay scheme. **(B)**. Antifungal assay as described by Kanini et al. (2013). In the figure from left to right: (1) *Fusarium oxysporum*, (2) *Gaeumannomyce*s sp., (3) *Colletotrichum* sp. 24C, (4) *Colletotrichum* sp. 26B, (5) *Phomosis* sp. DC1B, (6) *Rhizoctonia solani*, (7) *Fusarium* sp. DC9, (8) *Fusarium* sp. DC13B, (9) *Ulocladium* sp., (10) Assay scheme.

In order to study the strength and range of antimicrobial activity, all three selected strains were tested by Kirby-Bauer well-diffusion assays, which indicated that *Streptomyces* strains A20 and 5.1 could inhibit the growth of a wide range of bacterial and fungal phytopathogens to varying degrees, whereas strain 7.1 was able to inhibit mainly fungal phytopathogens (Table 1). Strain A20 showed the wider range of activity, with antimicrobial effects against 20 out of 21 bacterial species tested, and against all fungal pathogens evaluated (Figure 2). All three *Streptomyces* strains were also tested against a collection of 48 pathogenic *B. glumae* isolates isolated from Colombian rice-growing areas. In these assays, *Streptomyces* strains A20 and 5.1 exhibited antimicrobial activities against 91.48 and 78.72% of pathogens tested, respectively. Remarkably most of the pathogenic *B. glumae* isolates were inhibited by *Streptomyces* A20.

### *Streptomyces* Strains Isolated From Colombian Soils Are Able to Promote Rice Growth in Several Rice Cultivars

**FIGURE 3:**
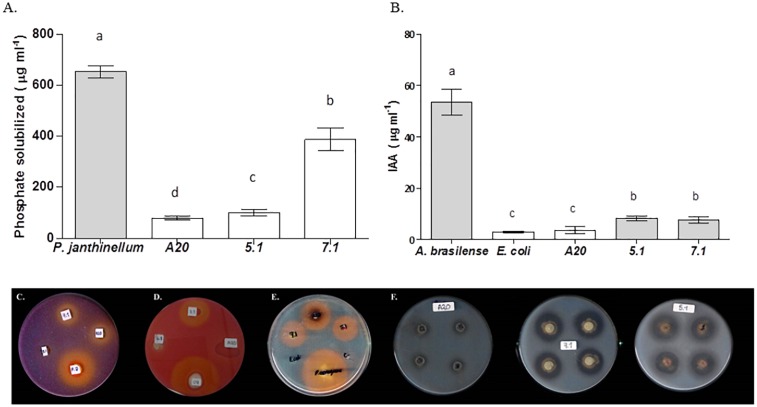
Evaluation of *Streptomyces* strains for key traits related to direct plant growth promotion. Bars correspond to **(A)** Phosphate solubilization assay or **(B)** Indoleacetic Acid production. The results are means of three values ± SD of three independent biological replicates. Means were compared with ANOVA analysis in combination with Tukey post-test. Means were considered statistically different when *p* < 0.05, and bars sharing a letter are not statistically different. **(C)** Qualitative phosphate solubilization assay, **(D)** Cellulase qualitative activity assay, **(E)** Siderophores qualitative production assay, **(F)** Protease qualitative production assay.

*In vitro* tests suggested that all three strains were able to produce siderophores, as well as proteolytic enzymes. All three strains also showed an ability to produce IAA and ACC deaminase, but none of the *Streptomyces* isolates could fix nitrogen. *Streptomyces* strain 7.1 was able to solubilize inorganic phosphate and showed a remarkable cellulolytic ability (Table 3 and Figure 3).

**Table 3:** Traits related with direct plant growth promotion.

Strain	Quantitative assay			Qualitative assay				
		
	BNF	Phosphate solubilization (μg mL^-1^)	IAA (μg mL^-1^)	Siderophore Production	ACC deaminase	Proteases (mm)	Cellulase activity (mm)	Ligninolytic activity
A20	UD	79.53 ± 7.43^a^	4.07 ± 1.83^a^	+	UD	12 ± 1.64	UD	UD
5.1	UD	101.54 ± 12.57^b^	7.98 ± 0.87^b^	+	UD	9.64 ± 1.24	UD	UD
7.1	UD	388.53 ± 43.66^c^	7.7 ± 1.3^b^	+	UD	17.43 ± 1.5	21.43 ± 2.06	UD


*Each assay was done three times with three biological replicates, and values represent their means ± standard deviation. One way-ANOVA (*p* < 0.05) was used to compare means. Values sharing a letter, within a column are not statistically different. UD, Undetectable activity. BNF, Biological Nitrogen Fixation. IAA, Indoleacetic Acid Production.*

**FIGURE 4:**
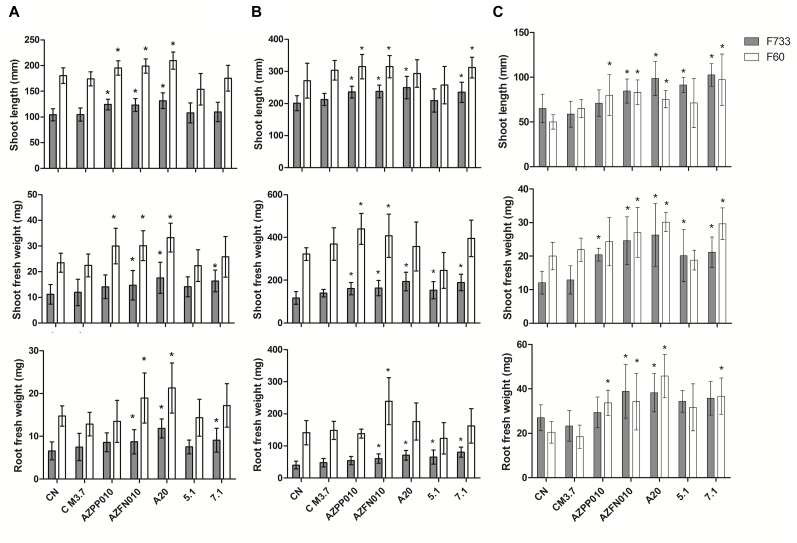
Plant-growth promotion experiments. Bars correspond to **(A)** Gnotobiotic assay, **(B)** Sterile Soil assay, and **(C)** Non-sterile soil assay. The results are means of twenty values ± SD, from two independent biological replicates. Means were compared with ANOVA analysis in combination with Tukey post-test, means were considered statistically different when *p* < 0.05. Bars with (^∗^) above are statistically different from the negative control. CN, Negative control. C M3.7, Medium control. AZPP010, *A. brasilense.* AZFN010, *A. chroococcum*.

Gnotobiotic inoculation experiments suggested that strain A20 was able to promote rice growth in comparison to non-inoculated plants, since it generated increases in shoot length and fresh weight in roots and plants of two different cultivars being however statistically significant only for cultivar F60 (Figure 4A). *Streptomyces* 7.1 was able to increase root and shoot fresh weight in the cultivar F733 and did not have any effect on rice cultivar F60. *Streptomyces* 5.1 did not show any increase in any of the evaluated parameters, in any of the cultivars tested. In soil experiments, *Streptomyces* 5.1 was able to improve the fresh weight of roots and shoots in cultivar 733, and strains A20 and 7.1 displayed the ability to significantly increase all the biometric parameters evaluated in cultivar F733 in comparison to the non-inoculated control (Figure 4B and Supplementary Figure S4).

Plant growth promotion experiments were performed both with sterile and non-sterile conditions in order to compare the performance of the three strains in such environments and foresee their behavior under natural conditions. In non-sterile soil, plants inoculated with *Streptomyces* A20 and *Streptomyces* 7.1 showed increases in their biometric parameters, with the same trend they showed in the gnotobiotic experiments. In contrast, strain *Streptomyces* 5.1 was able to increase plant length and fresh weight in plantlets from cultivar 733 (Figure 4C). Importantly, plantlets from non-sterile soils were smaller due to the presence of fungal strains, observed along the process (data not shown), which were most likely controlled by antifungal metabolites produced by isolates A20 and 5.1.

### *Streptomyces* A20, 5.1, and 7.1 Are Able to Colonize Two Different Rice Cultivars Under Gnotobiotic and Soil Conditions

Colonization experiments indicated that all *Streptomyces* strains tested were able to colonize the roots, since they were recovered from the rhizoplane in gnotobiotic experiments with roots from cultivars F733 and F60, even after 31 days post-inoculation (dpi) (Table 4 and Supplementary Material S2). *Streptomyces* A20 and *Streptomyces* 7.1 were also able to colonize the root endosphere, as they were recovered from a percentage of surface sterilized plants (80 and 20 %, respectively) grown in a gnotobiotic system.

**Table 4:** Bacterial counts in colonization experiments.

Strain/cultivar	Rhizoplane	Rhizosphere	Endosphere CFU (g dry weight) ^-1∗^		
			
		CFU (g soil)^-1^	Root^∗^	2 cm^∗^	Leaves^∗^
A20 (733)	+	1.66 × 10^6^ ± 0.21 a	1.65 × 10^2^ ± 0.18 a	1 × 10^4^	–
A20 (60)	+	1.3 × 10^6^ ± 0.43 a	3.43 × 10^2^± 0.40 a	1 × 10^2^	–
7.1 (733)	+	4.23 × 10^6^ ± 0.68 c	4.93 × 10^2^ ± 0.3 a	–	–
7.1 (60)	+	7.8 × 10^5^ ± 1.17 a,b	1.7 × 10^3^ ± 0.172 b	–	–
5.1 (733)	+	2.13 × 10^6^ ± 0.32 a	–	–	–
5.1 (60)	–	4.9 × 10^4^ ± 2.8 b	–	–	–


*Numbers represent means ± Standard Deviation for three biological replicates for each strain, with five plants per replicate. Different letters within a column indicate that values are statistically different, according to one way-ANOVA test (*p* < 0.05). ^∗^Endophytic colonization assay was performed under gnotobiotic condition.*

**FIGURE 5:**
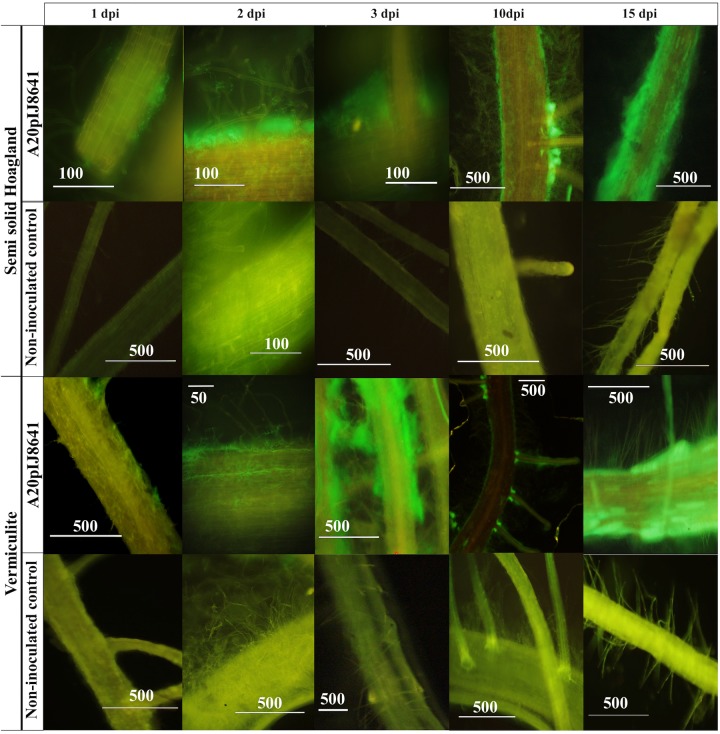
Microscopic visualization of rice root plants (cultivar F733) colonized with *Streptomyces* A20 EGFP mutant. This experiment was performed under gnotobiotic conditions with a non-inoculated control and an EGFP tagged strain (A20 pIJ8641), both in Semisolid Hoagland media and Vermiculite. Numbers above indicate days post inoculation (dpi), and numbers on bars correspond to the scale in micrometers.

An *egfp* tagged mutant was generated for strain A20 and colonization experiments were performed. Our results indicated that *egfp* expressing strain strongly associated with root hairs (Figure 5). Also, our experiments suggested that *Streptomyces* A20 first established itself in the main root and on the surface of the rice seeds (1 dpi, Figure 5), followed by the formation of micro-colonies between 2 and 3 dpi, and the colonization of secondary roots (Figure 5). After 10 days of growth, *Streptomyces* A20 had colonized the points of emergence for secondary roots and the surface of the main and secondary roots. After 15 days an overall colonization from the surface of the root is observed both in vermiculite and semisolid Hoagland media.

### *Streptomyces* A20 Produces Several Hydrophilic Charged Compounds

**FIGURE 6:**
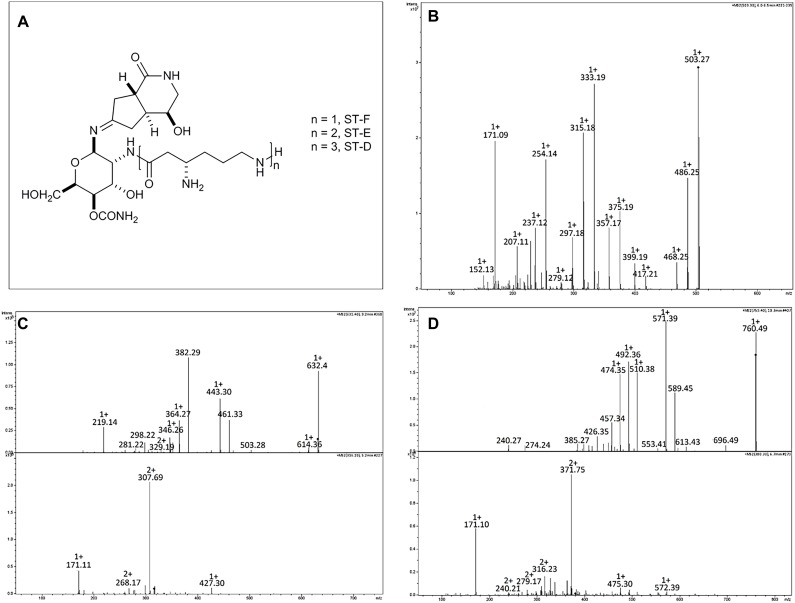
Structure and HPLC/ESI-MS analysis of the metabolites produced by strain *Streptomyces* A20. **(A)** Structures of the streptothricin-class antibiotics. MS/MS Spectra for **(B)** Streptothricin F (m/z 503.3), **(C)** Streptothricin D (m/z 631.4), and **(D)** Streptothricin E (m/z 759.5). For Streptothricins D and E lower panel corresponds to the MS/MS analysis of the doubly charged ion species for each compound. Analyzed compounds were obtained from fraction 16 collected in the RP-HPLC from the original fraction 32.

Strain A20 was chosen for further characterization of antimicrobial metabolites. Bioassay-guided extraction of A20 spent supernatants with solvents of increasing-polarity suggested that the antimicrobial compounds produced by this strain are hydrophilic ionic compounds since they remain in the aqueous fraction after the butanol extraction (Supplementary Figure S3). Ultrafiltration assays followed by antimicrobial activity assays indicated that the antimicrobial compounds present in residual aqueous fractions have sizes below 3 kDa (Table 5). Cation-exchange chromatography generated three more fractions with antimicrobial activity (namely fractions 21, 32, and 33), which were further submitted to ion-pairing chromatography in order to obtain defined peaks with antimicrobial activity. This technique allowed the resolution of 2 identical peaks in fractions 32 and 33, and one single peak in fraction 21, which possessed antibacterial activity. All the fractions were subsequently subjected to mass spectrometry analysis. Results have indicated that strain A20 produces three compounds with same spectra patterns and *m/z* values (503.3, 631.4, and 759.4), to those reported for Streptothricin F, D and E, as suggested by comparison of their mass spectra (Figure 6) (Ji et al., 2007; Maruyama and Hamano, 2009). Characterization of the streptothricin D was confirmed as follows: the MS/MS spectra for the parent ion at *m/z* 759.4 [M+H]^+^, proposed as the pseudomolecular ion for Streptothricine D, presented daughter ions at *m/z* 589 [M-L_3_]^+^, 571[M-L_3_-H_2_O], 554[M-L_3_-H_2_O-NH_3_]^+^, 553 [M-L_3_-H_2_O-H_2_O]^+^, and 510 [M-L_3_-H_2_O-H_2_O-CONH_2_]^+^ where the L_3_ represents the chain of three lysine residues of Streptothricin D, as suggested previously (Ji et al., 2007, 2008, 2009). These analyses were also applied for Streptothricin E and F, indicating the presence of an ion *m/z* 171, a signature peak characteristic of streptolidine lactam for all Streptothricins (Figure 6) (Ji et al., 2009).

**Table 5:** Ultrafiltration analyses for antimicrobial compounds produced by A20.

Fraction	*P. fuscovaginae UPB0736*	*B. glumae* AU6208
Aqueous phase (FAB)	+	+
<3 kDa	+	+
3–10 kDa	–	–
>10 kDa	–	–


*Ultrafiltration was performed with Amicon filters, by centrifuging at 1300 rpm × 30–40 min. Each fraction was ultrafiltrated, and the retentate was washed and centrifuged twice to avoid aggregate formation. (+) Positive antagonist activity (-) antagonist activity no detectable.*

## Discussion

High densities of antagonistic *Streptomyces* sp. have been associated with plant disease suppression in many soils, and thus biocontrol via bioinoculants is today regarded as a promising alternative for the management of bacterial diseases. These potential biocontrol bacteria can combine several mechanisms to inhibit pathogens in soil; which include nutrient competition, production of degradative enzymes, nitrous oxide production and quorum quenching (Kinkel et al., 2012). *Streptomyces* spp. have been widely recognized for their potential for controlling fungal pathogens, including *Fusarium moniliforme, Fusarium oxysporum f. sp. ciceri, Rhizoctonia solani, R. bataticola, Magnaporthe grisea*, and *Magnaporthe oryzae* (Emmert and Handelsman, 1999; Samac and Kinkel, 2001; Taechowisan et al., 2003; Tian et al., 2007; Zarandi et al., 2009; Gopalakrishnan et al., 2011; Kanini et al., 2013), as well as rice bacterial pathogen *Xanthomonas oryzae* pv. *oryzae* (Hastuti et al., 2012). Furthermore, diversity of secondary metabolite production among *Streptomyces* has supported the formulation of inoculants for biocontrol like Mycostop^®^ (Verdera, Finland) and Actinovate (Natural Industries, Incorporated, United States), based on *S. griseoviridis* K61 and *S. lidicus* WYEC 108, respectively.

Biocontrol agent selection is not a simple task due to the diversity of agents and interactions with the host plant (Mota et al., 2017). Efficient selection strategies have been proposed in order to increase the possibility of identifying organisms that can be produced in a large scale and maintain their viability and efficiency for extended periods of time (Schisler et al., 1997; Köhl et al., 2011). This study presents a systematic assessment of *Streptomyces* strains for development of bioinoculants able to promote plant growth and control rice phytopathogens like *B. glumae* and *P. fuscovaginae*. Our approach included: (1) Screening for actinobacterial isolates from the rice rhizosphere, favoring selection of bacteria from the same ecological niche; (2) Screening for PGPR and biocontrol properties *in vitro*, (3) Taxonomical identification for isolates with broader antimicrobial activity, (4) Assessment of plant growth promotion and colonization, *in vivo*, and (4) Antimicrobial compounds identification.

Sixty actinobacterial-like isolates were obtained from Colombian soils upon enrichment with CaCO_3_, but only three of them (A20, 5.1 and 7.1) showed antibacterial activity against pathogenic bacterial strains of both *B. glumae* and *P. fuscovaginae.* Taxonomical analyses based on 16S rRNA confirmed that all three strains belong to the genus *Streptomyces*, and MLST results suggested that these isolates might represent new species within the genus. Evolutionary distance analyses between concatenated loci from isolate A20 and type strain *S. racemochromogenes* DSM 40194 suggested that *Streptomyces* A20 is likely to be a new member of this species clade.

Those selected isolates were further studied, regarding their antimicrobial potential, plant growth promotion and plant colonization. Despite their common origin (rice rhizosphere), all strains showed a different spectrum of action, being *Streptomyces* A20 able to inhibit growth for a broader range of bacterial and fungal phytopathogens. When tested against 48 *B. glumae* pathogenic isolates, most of them were also inhibited by *Streptomyces* A20, which indicated that this isolate could be potentially used, alone or in combination with other strains to control BPB.

Production of broad-spectrum antimicrobials has been reported for plant-associated *Streptomyces* species, supporting their use to control fungal and nematode phytopathogens (Emmert and Handelsman, 1999; Samac and Kinkel, 2001; Taechowisan et al., 2003; Tian et al., 2004; Zarandi et al., 2009). Furthermore, biogeographic studies about the ecology of *Streptomyces* have hypothesized that *Streptomyces* with broad and highly potent inhibitory phenotypes might be competitive ‘hot spots,’ which have selected for populations that are useful inhibitors of resource competitors, which may be the case for rice rhizosphere (Schlatter and Kinkel, 2014; Behie et al., 2016).

The three selected strains were tested *in vitro* for properties that are known to be important for plant growth promoting activities of bacteria, such as the production of siderophores, solubilization of inorganic phosphate, phytohormone levels etc. Overall, these suggested that all three strains can solubilize phosphate and produce siderophores, proteases, and IAA, which are also typical of plant-associated Actinobacteria, and have been related to plant-growth promotion (Nimnoi et al., 2010; Bouizgarne and Aouamar, 2014; Carvalho et al., 2017).

Many biocontrol bacteria are only capable of preventing or limiting the establishment of soilborne pathogens after colonizing plant roots, as root colonization is necessary for the suppression of root pathogens (Mota et al., 2017). As a result, several studies have been directed at identifying the different bacterial traits that may contribute to their *rhizospheric competence* (De Weert and Bloemberg, 2006). Since *in vitro* experiments revealed that *Streptomyces* A20, 5.1 and 7.1 strains carry several features important for biocontrol and plant growth promotion, we were interested in assessing plant growth promotion and colonization *in vivo*, using two cultivars of rice. Plant growth promotion experiments were performed both with sterile and non-sterile conditions in order to compare the performance of these strains in such environments and foresee their behavior under natural conditions. Our results indicated that in non-sterile soil, plants inoculated with *Streptomyces* A20 and *Streptomyces* 7.1 showed significant increases in their biometric parameters, with the same trend they showed in the gnotobiotic experiments. Furthermore, colonization experiments indicated that all *Streptomyces* strains tested were able to colonize the roots, since they were able to prevail in close association with roots, in two different cultivars, even after 31 days post-inoculation (dpi).

Interestingly, *Streptomyces* A20 and *Streptomyces* 7.1 were also able to colonize the root endosphere, as they were recovered from a percentage of surface sterilized plants (80 and 20%, respectively), grown in a gnotobiotic system. This last trend was not observed for plants grown in soil indicating that it might be due to the growth conditions, as well as possible competition against the other microbiota present in the soil and associated to the plant. These results are consistent with similar findings reported for other Actinobacteria, including *Streptomyces* strains (*S. cyaneus, S. lanatus*, and *S. galilaeus)*, which were also recovered from the endosphere of the roots (Tian et al., 2004, 2007; Knief et al., 2012). Field experiments with more extended growth periods may be required to determine if these strains can colonize the endosphere under natural conditions.

In order to monitor the rice colonization process by strain *Streptomyces* A20, colonization experiments were performed with an *egfp* tagged mutant. Our results indicated that *egfp* expressing strain strongly associated with root hairs and experiments in inoculated and non-inoculated plants showed an increase in root-hair formation, which may be related to the plant growth promotion observed in the gnotobiotic experiments.

Antimicrobial compound identification is convenient when developing biocontrol products. In this study, several chromatographic techniques were used to purify antimicrobial compounds produced by strain A20, that exhibited antibacterial activity against *B. glumae* AU6208 and *P. fuscovaginae* UPB0736. Results indicated that strain A20 produces three close related compounds, according to their similar spectra patterns (*m/z* values at 503.3, 631.4, and 759.4). The mass spectra and their MS/MS study allowed identifying those compounds as Streptothricin F, D and E. A typical streptothricin structure consists of a streptolidine, a carbamoyl-D-gulosamine and a β-lysine chain, which varies from one to six units in streptothricins F to A, respectively (Khokhlov and Shutova, 1972; Telesnina et al., 1973; Khokhlov, 1983). Streptothricins (STs) were the earliest reported antibiotics with a broad antimicrobial spectrum from actinomycetes and are found in approximately 10% of randomly collected soil actinomycetes. These compounds are also known as nourseothricins, racemomycins, and yazumycins, and the first member of this group, designated ST-F, was isolated from *Streptomyces lavendulae* in 1943 (Waksman, 1943; Maruyama and Hamano, 2009).

STs are known to inhibit protein biosynthesis in prokaryotic cells and strongly inhibit the growth of eukaryotes like fungi and have been suggested as a selection marker for a wide range of organisms including bacteria, yeast, filamentous fungi, and plant cells. A known example is Nourseothricin (CloNat), an effective selective agent for molecular cloning technologies in fungi and plants (Goldstein and McCusker, 1999; Kochupurakkal and Iglehart, 2013). Despite their wide range of antimicrobial activity, STs are not currently indicated for clinical or veterinary uses because of their inherent toxicity, including nephrotoxicity (Hartl et al., 1986). On the other hand, streptothricins are currently being tested as fungistatic agents in agriculture for the treatment of blast and other plant diseases (Goo et al., 1996). Lastly, STs have been registered as agrochemical fungicides in China (Kusumoto et al., 1982).

As indicated in the *Streptomyces* chemical database StreptomeDB (Lucas et al., 2013), streptothricins production has been reported for *Streptomyces ginlingensis, S. rochei, S. flavus, S. lavendulae, S. griseus, S. roseochromogenes*, and *S. noursei*. Maruyama and Hamano reported an extended study describing the genetic cluster responsible of the streptothricin synthesis in *S. rochei* NBRC12908. Interestingly, this cluster includes a self-resistance gene encoding a ST acetyltransferase (SAT). In addition degenerate primers were proposed as a tool to identify SAT-related immunity genes using highly conserved amino acid sequences of SATs identified in ST-resistant microorganisms (Maruyama et al., 2012). We did not obtain amplification for a potential SAT locus using genomic DNA from *Streptomyces* A20 (data not shown); this may indicate that this locus in strain A20 differs at sequence level from those reported so far, although it is also possible that strain A20 possesses a different mechanism for ST tolerance.

Plant growth promotion, colonization abilities and antimicrobial activities shown by strain *Streptomyces* A20 suggest that this strain could be used for plant growth promotion and biocontrol in rice. One significant advantage for this isolate is that it was initially recovered from rice sown soils, and thus it fulfills the premise that microorganisms chosen for inoculation should ideally be selected from local ecological niches and re-inoculated into the same environments, to ensure the desired benefits. Field experiments are necessary to further verify the effects on rice plants under natural growth conditions. Further studies will allow establishing the relationship between application of strain *Streptomyces* A20 and plant disease suppression, as well as the spatial-temporal dynamics of the pathogens and antagonists in soil (Johnson and Dileone, 1999).

## Author Contributions

ZS-M, DV-V, and DV-M performed the experiments for bacterial isolation, identification and antimicrobial screening and plant experiments. LC and FR designed chemical fractionation schemes for the elucidation of bioactive compounds and data analyses for all chromatographic techniques. CG and GD developed ultrafiltration studies, chromatography and mass spectrometry experiments for compound elucidation and provided advice about data analyses. ZS-M, NM-S, LC, FR, GD, and VV contributed to the conception and design of the study. NM-S was the leader of the project. All authors contributed to the manuscript revision, read and approved the submitted version.

## Conflict of Interest Statement

The authors declare that the research was conducted in the absence of any commercial or financial relationships that could be construed as a potential conflict of interest.

## Footnotes

1 http://pubmlst.org/Streptomyces/

## Supplementary Material

Figure S1 The figure shows the plate count technique used for the determination of Colony-Forming Units of *Streptomyces* isolates **(A)** and the macroscopic and microscopic characterization of the selected *Streptomyces* strains **(B)**.

Figure S2 Rice colonization assays. Two independent assays were performed under gnotobiotic and soil conditions, using three biological replicates for each strain, with five plants per replicate in two rice cultivars (F60 and F733). Bacterial strains were re-isolated from the rhizoplane by cutting and washing 2 cm segments of each plant root and placing the excised root segments on the surface of ISP3 plates. To assess the ability of each strain to colonize the rhizoplane, samples were macerated and serially diluted and plated on ISP3 plates. Note that strain *Streptomyces* A20 was able to colonize both cultivars. To verify endophytic colonization, inoculated plants were surface sterilized and each plant was then excised in the root, plant shoot and leaves, and each part was macerated with a sterile pestle. Each suspension was then serially diluted and plated in ISP3 plates, and surface sterilization was regarded as optimum when no growth was observed after placing the processed plants on nutrient agar plates. This experiment suggests that strains A20 and 7.1 are able to colonize the endosphere of both cultivars. Furthermore, microscopy of the treated roots, stained with Lactophenol blue suggests the presence of *Streptomyces* A20 mycelium on and around the surface of and adventitious root (see arrows).

Figure S3 Bioassay-guided fractionation of A20 antibacterial compounds. **(A)** Fractionation Scheme. **(B)** Antimicrobial assays in *B. glumae* AU6208 and *P. fuscovaginae* UPB0736. The red numbers in the fractionating scheme **(A)** correspond to the fractions tested in the Kirby-Bauer test shown in **(B)**. Control plates include Gentamycin (Gm) as positive control for antimicrobial activity, and Water (W) as negative control. To exclude any effect from the media, media, M3.7 was processed in the same way, and fractions 3, 4, 5, and 6 were tested in control plates.

Figure S4 Picture of inoculated rice plants (Cultivar 733) with *Streptomyces* spp. strains vs. controls. *Streptomyces* 5.1 was able to improve the fresh weight of roots and shoots in cultivar 733, and strains A20 and 7.1 displayed the ability to significantly increase all the biometric parameters evaluated in cultivar F733 in comparison to the non-inoculated control. *Azotobacter chroococcum* was used as positive control for plant growth promotion (AC).

Table S1 Microbial strains and primers used in this study.

## Abbreviations

ACC: 1-aminocyclopropane-1-carboxylate
BPB: Bacterial panicle Blight
CMC: carboxymethylcelulose
IAA: Indoleacetic Acid
LC-MS: liquid chromatography–mass spectrometry
MLST: Multilocus Sequence Typing
PGPB: Plant-Growth Promoting Bacteria
SBR: Sheath Brown Rot
