# Supplementary figures and images for: Plant-Growth Promotion and Biocontrol Properties of Three Streptomyces spp. Isolates to Control Bacterial Rice Pathogens
Source: Front Microbiol. 2019 Feb 25;10:290. doi: 10.3389/fmicb.2019.00290 (PMC6398372; doi:10.3389/fmicb.2019.00290)

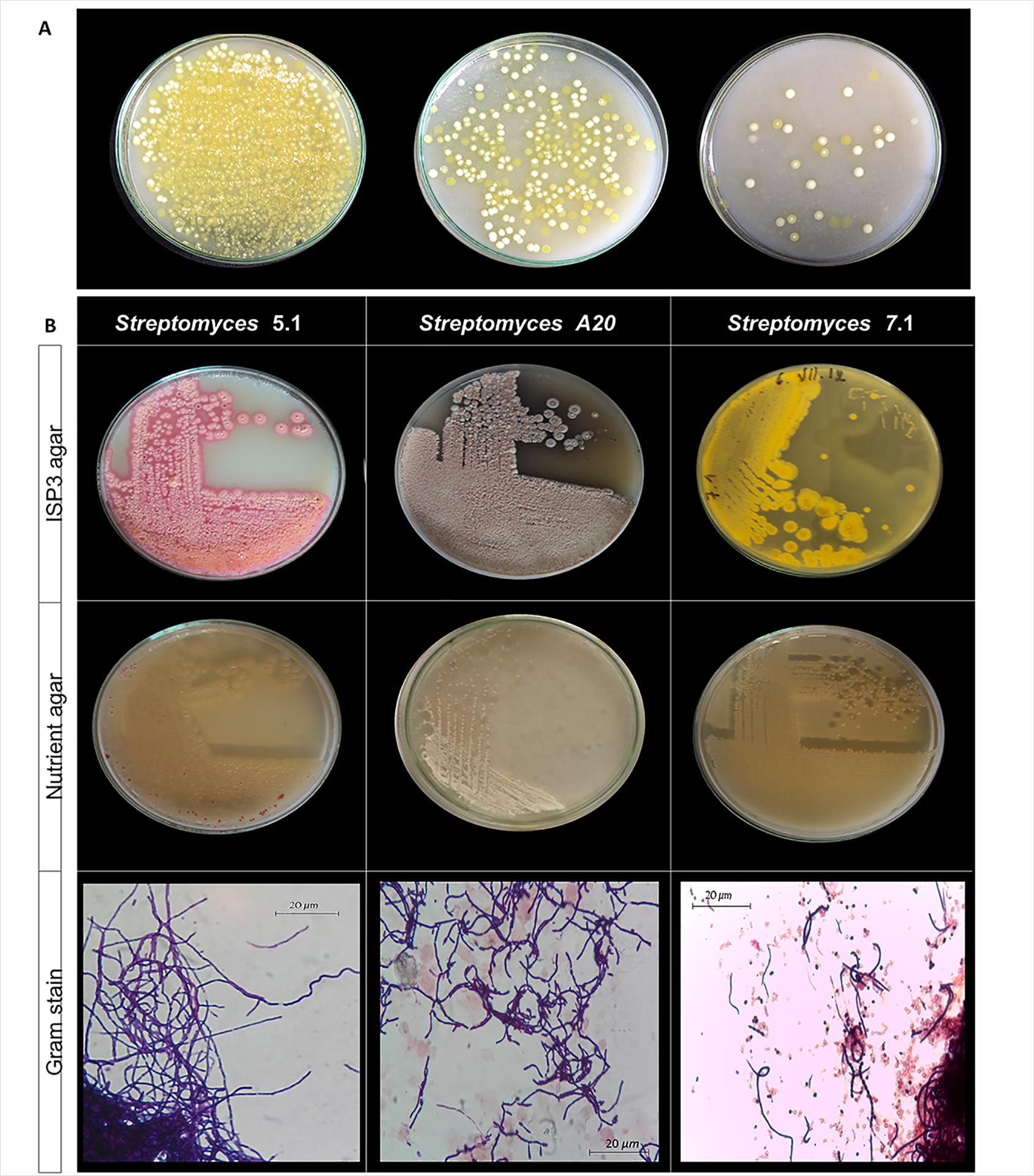

Supplement: Figure S1 — The figure shows the plate count technique used for the determination of Colony-Forming Units of Streptomyces isolates (A) and the macroscopic and microscopic characterization of the selected Streptomyces strains (B). [file Image_1.TIF]

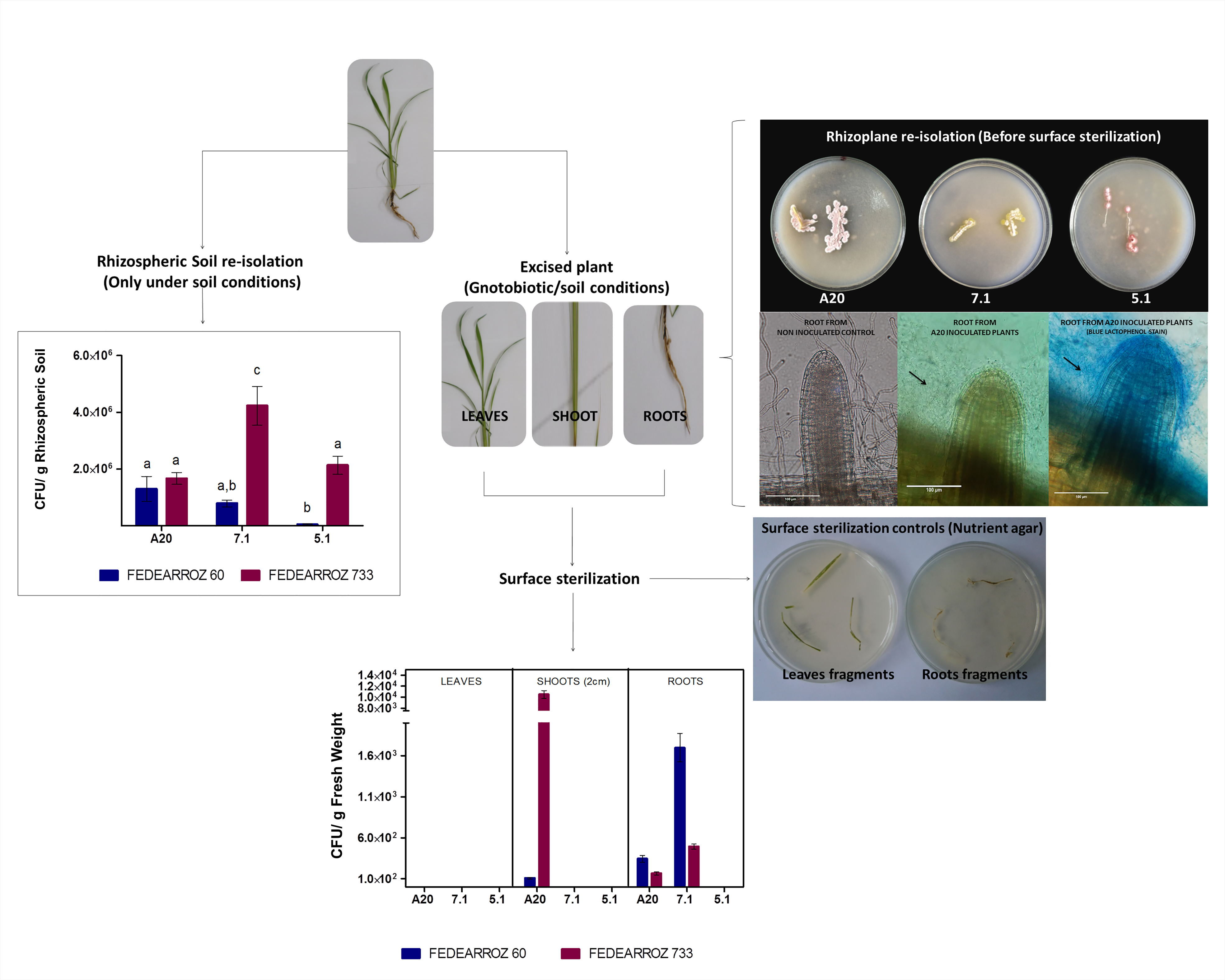

Supplement: Figure S2 — Rice colonization assays. Two independent assays were performed under gnotobiotic and soil conditions, using three biological replicates for each strain, with five plants per replicate in two rice cultivars (F60 and F733). Bacterial strains were re-isolated from the rhizoplane by cutting and washing 2 cm segments of each plant root and placing the excised root segments on the surface of ISP3 plates. To assess the ability of each strain to colonize the rhizoplane, samples were macerated and serially diluted and plated on ISP3 plates. Note that strain Streptomyces A20 was able to colonize both cultivars. To verify endophytic colonization, inoculated plants were surface sterilized and each plant was then excised in the root, plant shoot and leaves, and each part was macerated with a sterile pestle. Each suspension was then serially diluted and plated in ISP3 plates, and surface sterilization was regarded as optimum when no growth was observed after placing the processed plants on nutrient agar plates. This experiment suggests that strains A20 and 7.1 are able to colonize the endosphere of both cultivars. Furthermore, microscopy of the treated roots, stained with Lactophenol blue suggests the presence of Streptomyces A20 mycelium on and around the surface of and adventitious root (see arrows). [file Image_2.TIF]

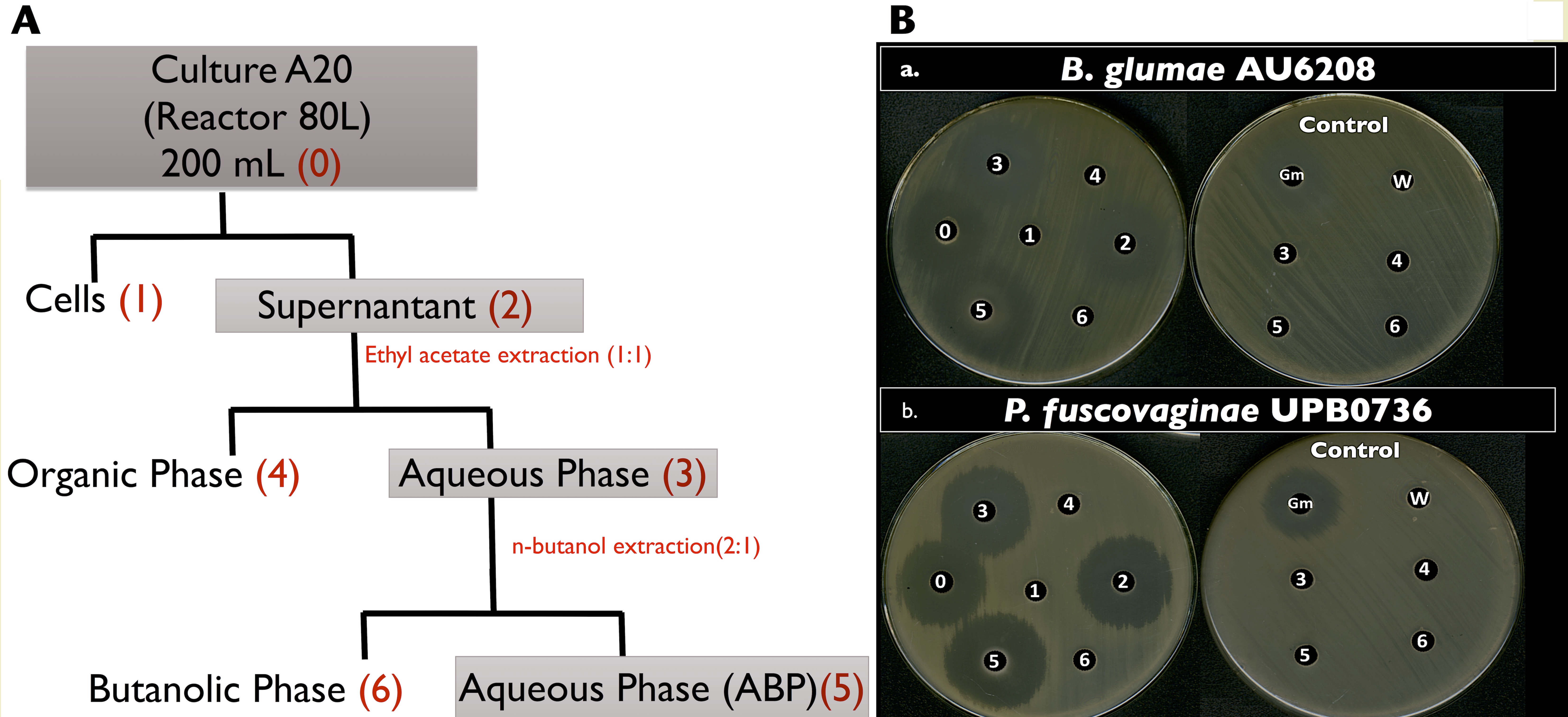

Supplement: Figure S3 — Bioassay-guided fractionation of A20 antibacterial compounds. (A) Fractionation Scheme. (B) Antimicrobial assays in B. glumae AU6208 and P. fuscovaginae UPB0736. The red numbers in the fractionating scheme (A) correspond to the fractions tested in the Kirby-Bauer test shown in (B). Control plates include Gentamycin (Gm) as positive control for antimicrobial activity, and Water (W) as negative control. To exclude any effect from the media, media, M3.7 was processed in the same way, and fractions 3, 4, 5, and 6 were tested in control plates. [file Image_3.jpg]

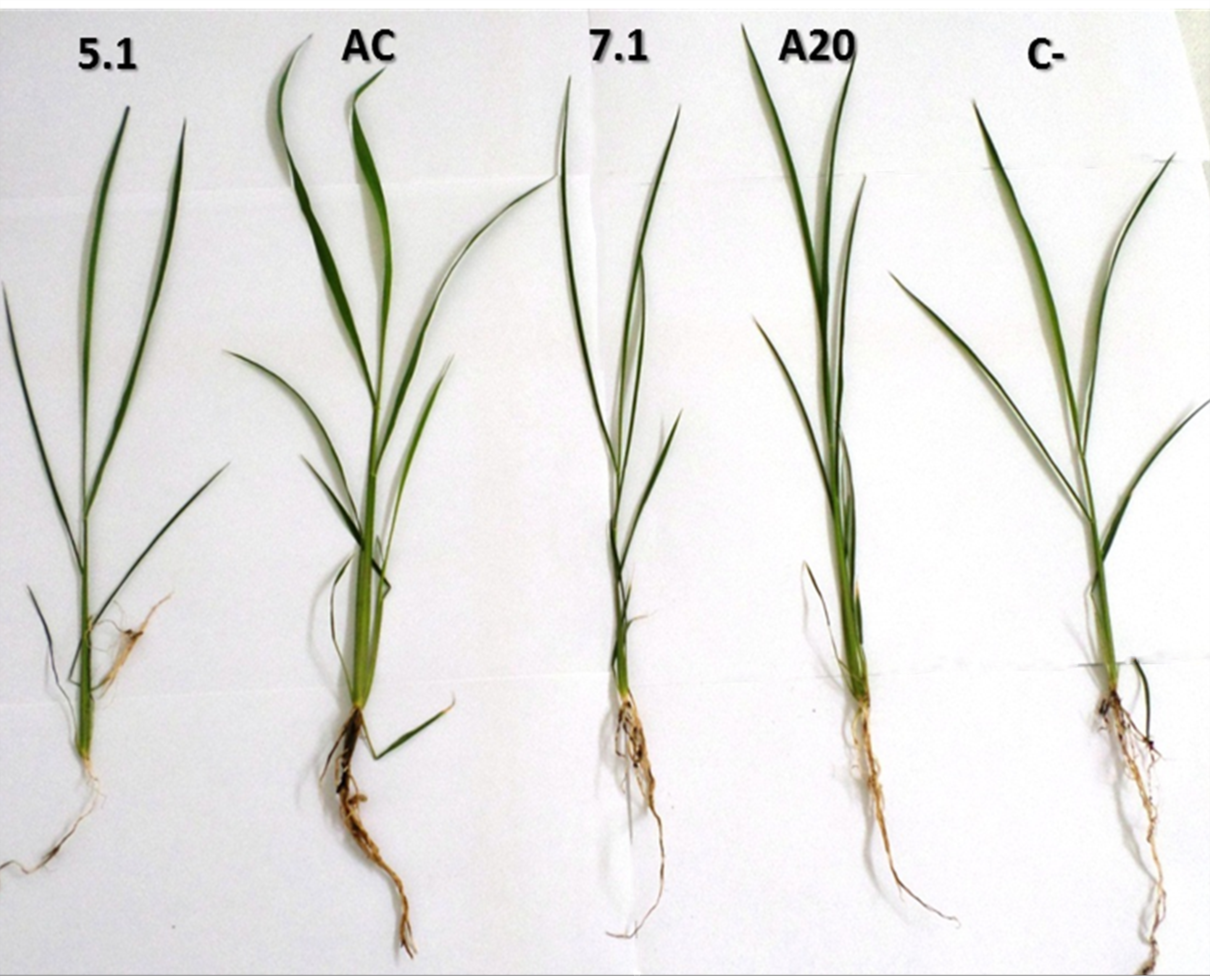

Supplement: Figure S4 — Picture of inoculated rice plants (Cultivar 733) with Streptomyces spp. strains vs. controls. Streptomyces 5.1 was able to improve the fresh weight of roots and shoots in cultivar 733, and strains A20 and 7.1 displayed the ability to significantly increase all the biometric parameters evaluated in cultivar F733 in comparison to the non-inoculated control. Azotobacter chroococcum was used as positive control for plant growth promotion (AC). [file Image_4.TIF]
