# Supplementary material for: Plant-Growth Promotion and Biocontrol Properties of Three Streptomyces spp. Isolates to Control Bacterial Rice Pathogens
Source: Front Microbiol. 2019 Feb 25;10:290. doi: 10.3389/fmicb.2019.00290 (PMC6398372; doi:10.3389/fmicb.2019.00290)
Supplement: Table S1 — Microbial strains and primers used in this study. [file Table_1.docx]

**Supplementary Table 1. Microbial strains and primers used in this study.**

| \| **STRAIN** \| **CHARACTERISTICS** \| **SOURCE** \| \| --- \| --- \| --- \| \| **BACTERIAL STRAINS** \|  \|  \| \| ***Streptomyces strains*** \|  \|  \| \| *Streptomyces* A20 \| Rice rhizosphere isolate \| This study \| \| *Streptomyces* 5.1 \| Rice rhizosphere isolate \| This study \| \| *Streptomyces* 7.1 \| Rice rhizosphere isolate \| This study \| \| **BACTERIAL STRAINS FOR ANTIMICROBIAL ASSAYS** \|  \|  \| \| *Acidovorax avenae* 4008-2 \| Rice pathogen \| CIAT (Unpublished) \| \| *Acinetobacter baumannii*  ATCC 19606 \| Human pathogen \| ATCC \| \| *Bacillus* sp*. C636* \| Native strain \| IBUN strain collection \| \| *Bacillus subtilis* ATCC 21556 \| Commercial strain \| ATCC \| \| *Burkholderia glumae* 320012 \| Phytopathogen \| CIAT (Unpublished) \| \| *Burkholderia. glumae* CIAT 4026 \| Phytopathogen \| CIAT (Unpublished) \| \| *Burkholderia glumae* AU6208 \| Clinical isolate \| (Weinberg et al., 2007) \| \| *Burkholderia gladioli* CIAT 3704-1 \| Rice pathogen \| CIAT (Unpublished) \| \| *B. gladioli* CIAT 3962 \| Rice pathogen \| CIAT (Unpublished) \| \| *Chromobacterium violaceum* ATCC 31532 \| Commercial strain \| ATCC \| \| *Enterobacter aerogenes* 7ARG \| Commercial strain \| IBUN strain collection \| \| *Escherichia coli DH5α* \| Commercial strain \| GIBCO-BBL Life Technologies \| \| *Escherichia coli* ATCC 23724 \| Commercial strain \| ATCC \| \| *Escherichia coli* ATCC 25922 \| Commercial strain \| ATCC \| \| *Klebsiella pneumoniae* ATCC 700603 \| Multi-resistant strain \| ATCC \| \| *Pseudomonas putida* F117 pKRC12 \| QS-Biosensor strain \| (Riedel et al., 2001) \| \| *Pseudomonas aeruginosa* ATCC 27853 \| Commercial strain \| ATCC \| \| *Pseudomonas fuscovaginae* CIAT 3638-19 \| Phytopathogen \| CIAT (Unpublished) \| \| *Pseudomonas. fuscovaginae* CIAT 3668π3 \| Phytopathogen \| CIAT (Unpublished) \| \| *Pseudomonas fuscovaginae* UPB0736 \| Phytopathogen \| (Mattiuzzo et al., 2010) \| \| *Staphylococcus aureus* ATCC 25923 \| Human pathogen \| ATCC \| \| **FUNGAL STRAINS** \|  \|  \| \| *Colletotrichum* sp. *24C* (FIF024) \| Yam pathogen \| Yam research group – IBUN \| \| *Colletotrichum* sp. *26B* \| Yam pathogen \| Yam research group – IBUN \| \| *Fusarium* sp*. DC9 (*FIF025) \| Yam pathogen \| Yam research group – IBUN \| \| *Fusarium* sp. *DC 13B* \| Yam pathogen \| Yam research group – IBUN \| \| *Fusarium oxysporum* \| Clover pathogen \| Group study plant metabolic activities.  Department of Chemistry UNAL \| \| *Gaeumannomyces* sp*.* (FPR010) \| Rice pathogen \| Yam research group – IBUN \| \| *Penicillium janthinellum* FP010 \| Native strain \| (Scervino et al., 2010) \| \| *Phomopsis* sp*.*DC1B (FIF027) \| Yam pathogen \| Yam research group – IBUN \| \| *Rhizoctonia solani* (FPR011) \| Rice pathogen \| Yam research group – IBUN \| \| *Ullocladium* sp*.* 29E (FIF023) \| Yam pathogen \| Yam research group – IBUN \| \| **PLANT GROWTH PROMOTING STRAINS** \|  \|  \| \| *Azospirillum brasilense* AZPP010 \| Plant Growth Promoting Strain \| (Moreno and Rojas, 2008) \| \| *Azotobacter chroccoccum* AZFN010 \| Growth Promoting Strain \| (Moreno, 2011) \|  \| **Locus** \| **Primer** \| **Sequence (5´🡪 3´)** \| **Reference** \| \| --- \| --- \| --- \| --- \| \| 16S \| 16SA \| CCGTCGACGAGCTCAGAGTTTGATCCTGGCTCAG \| (Cui et al., 2001) \| \| 16S \| 16SB \| CCCGGGTACCAAGCTTAAGGAGGTGATCCAGCCGCA \| \| *gyrB* \| gyrBPF  gyrBPR \| GAGGTCGTGCTGACCGTGCTGCACGCGGGCGGCAAGTTCGGC  GTTGATGTGCTGGCCGTCGACGTCGGCGTCCGCCAT \| (Guo et al., 2008) \| \| *atpD* \| atpDPF  atpDPR \| GTCGGCGACTTCACCAAGGGCAAGGTGTTCAACACC  GTGAACTGCTTGGCGACGTGGGTGTTCTGGGACAGGAA \| \| *recA* \| recAPF  recAPR \| CCGCRCTCGCACAGATTGAACGSCAATTC  GCSAGGTCGGGGTTGTCCTTSAGGAAGTTGCG \| \| *trpB* \| trpBPF trpBPR \| GCGCGAGGACCTGAACCACACCGGCTCACACAAGATCAACA  TCGATGGCCGGGATGATGCCCTCGGTGCGCGACAGCAGGC \| \| *rpoB* \| rpoBPF  rpoBPR \| GAGCGCATGACCACCCAGGACGTCGAGGC  CCTCGTAGTTGTGACCCTCCCACGGCATGA \| |  |  |
| --- | --- | --- | --- | --- | --- | --- | --- | --- | --- | --- | --- | --- | --- | --- | --- | --- | --- | --- | --- | --- | --- | --- | --- | --- | --- | --- | --- | --- | --- | --- | --- | --- | --- | --- | --- | --- | --- | --- | --- | --- | --- | --- | --- | --- | --- | --- | --- | --- | --- | --- | --- | --- | --- | --- | --- | --- | --- | --- | --- | --- | --- | --- | --- | --- | --- | --- | --- | --- | --- | --- | --- | --- | --- | --- | --- | --- | --- | --- | --- | --- | --- | --- | --- | --- | --- | --- | --- | --- | --- | --- | --- | --- | --- | --- | --- | --- | --- | --- | --- | --- | --- | --- | --- | --- | --- | --- | --- | --- | --- | --- | --- | --- | --- | --- | --- | --- | --- | --- | --- | --- | --- | --- | --- | --- | --- | --- | --- | --- | --- | --- | --- | --- | --- | --- | --- | --- | --- | --- | --- | --- | --- | --- | --- | --- | --- | --- | --- | --- | --- | --- | --- | --- | --- | --- | --- |
